# Melatonin: the placental antioxidant and anti-inflammatory

**DOI:** 10.3389/fimmu.2024.1339304

**Published:** 2024-02-01

**Authors:** Tyana T. Joseph, Viviane Schuch, Daniel J. Hossack, Rana Chakraborty, Erica L. Johnson

**Affiliations:** ^1^ Microbiology, Biochemistry and Immunology, Morehouse School of Medicine, Atlanta, GA, United States; ^2^ Department of Pediatric and Adolescent Medicine, Mayo Clinic College of Medicine and Science, Rochester, MN, United States

**Keywords:** melatonin, antioxidant, anti-inflammatory, placenta, pregnancy, HCMV, oxidative stress, NLRP3 inflammasome

## Abstract

Melatonin (N-acetyl-5-methoxytryptamine) is an indolamine hormone with many physiological and biological roles. Melatonin is an antioxidant, anti-inflammatory, free radical scavenger, circadian rhythm regulator, and sleep hormone. However, its most popular role is the ability to regulate sleep through the circadian rhythm. Interestingly, recent studies have shown that melatonin is an important and essential hormone during pregnancy, specifically in the placenta. This is primarily due to the placenta’s ability to synthesize its own melatonin rather than depending on the pineal gland. During pregnancy, melatonin acts as an antioxidant and anti-inflammatory, which is necessary to ensure a stable environment for both the mother and the fetus. It is an essential antioxidant in the placenta because it reduces oxidative stress by constantly scavenging for free radicals, i.e., maintain the placenta’s integrity. In a healthy pregnancy, the maternal immune system is constantly altered to accommodate the needs of the growing fetus, and melatonin acts as a key anti-inflammatory by regulating immune homeostasis during early and late gestation. This literature review aims to identify and summarize melatonin’s role as a powerful antioxidant and anti-inflammatory that reduces oxidative stress and inflammation to maintain a favorable homeostatic environment in the placenta throughout gestation.

## 1 Introduction

Throughout gestation, melatonin (N-acetyl-5-methoxytryptamine) is a powerful hormone with many physiological and biological roles that ensure a stable environment for both the mother and the fetus. Melatonin is an antioxidant, anti-inflammatory, free radical scavenger, circadian rhythm regulator, and sleep hormone (1, 2). A homeostatic balance between reactive oxygen species (ROS) and antioxidants is needed during pregnancy to maintain a stable and healthy placenta. Without this balance, oxidative stress can occur in the placenta, allowing adverse conditions like pre-eclampsia, preterm birth, and intrauterine growth restriction (IUGR) to occur (3–5). Melatonin is an essential antioxidant in the placenta that reduces oxidative stress during gestation (6, 7). Melatonin is a lipophilic, hydrophilic indolamine that can rapidly cross the placenta and diffuse into cells (1, 8). In addition, this hormone is endogenously produced in both the ovaries and the placenta, which results in increased levels of systemic melatonin in pregnant women compared to non-pregnant women (9, 10). However, melatonin’s mechanistic role and signaling pathways during pregnancy are largely unknown (1, 11–13). In this review, we will provide in-depth insight into melatonin’s role in the placenta as an anti-inflammatory and antioxidant during inflammation, oxidative stress, and viral infection.

## 2 The placenta

### 2.1 Overview

The placenta is a multifaceted, temporary organ with numerous biological functions largely considered endocrinologic and immunologic (14). The critical roles of the placenta include facilitating embryonic implantation into the uterine wall, promoting fetal growth, and maintaining maternal–fetal tolerance (15). In addition, this organ removes harmful waste products and carbon dioxide from the fetal circulation, provides nutrients and oxygen, and envelopes the fetus in a protective immunologic membrane throughout gestation (15–17). The umbilical cord transfers blood through the placenta to supply the fetus with adequate oxygen and nutrients for survival throughout pregnancy (18). This exchange occurs without the fetal blood and the maternal blood intermingling (17, 18). Hormones produced by the placenta include estrogen and progesterone, which promote the expansion of the uterus to accommodate the growing fetus and placenta (19). Other key immunoprotective roles include facilitating the passive transfer of IgG from the maternal to the fetal circulation and affording protection against invasive pathogens in intrauterine and postnatal life (20, 21).

### 2.2 Development

The formation of the placenta begins after the fertilized egg is implanted into the uterus, which occurs approximately 8 to 10 days after conception (15, 17). The placenta will gradually grow during the first 3 months of pregnancy and then increase in size corresponding to the uterus after 4 months (15, 17). There are several layers and sublayers of tissue that constitute the multifunctional placenta. This review will primarily focus on a sublayer in the chorion layer, namely, the trophoblast, and the decidual layer of the placenta.

**Figure 1:**
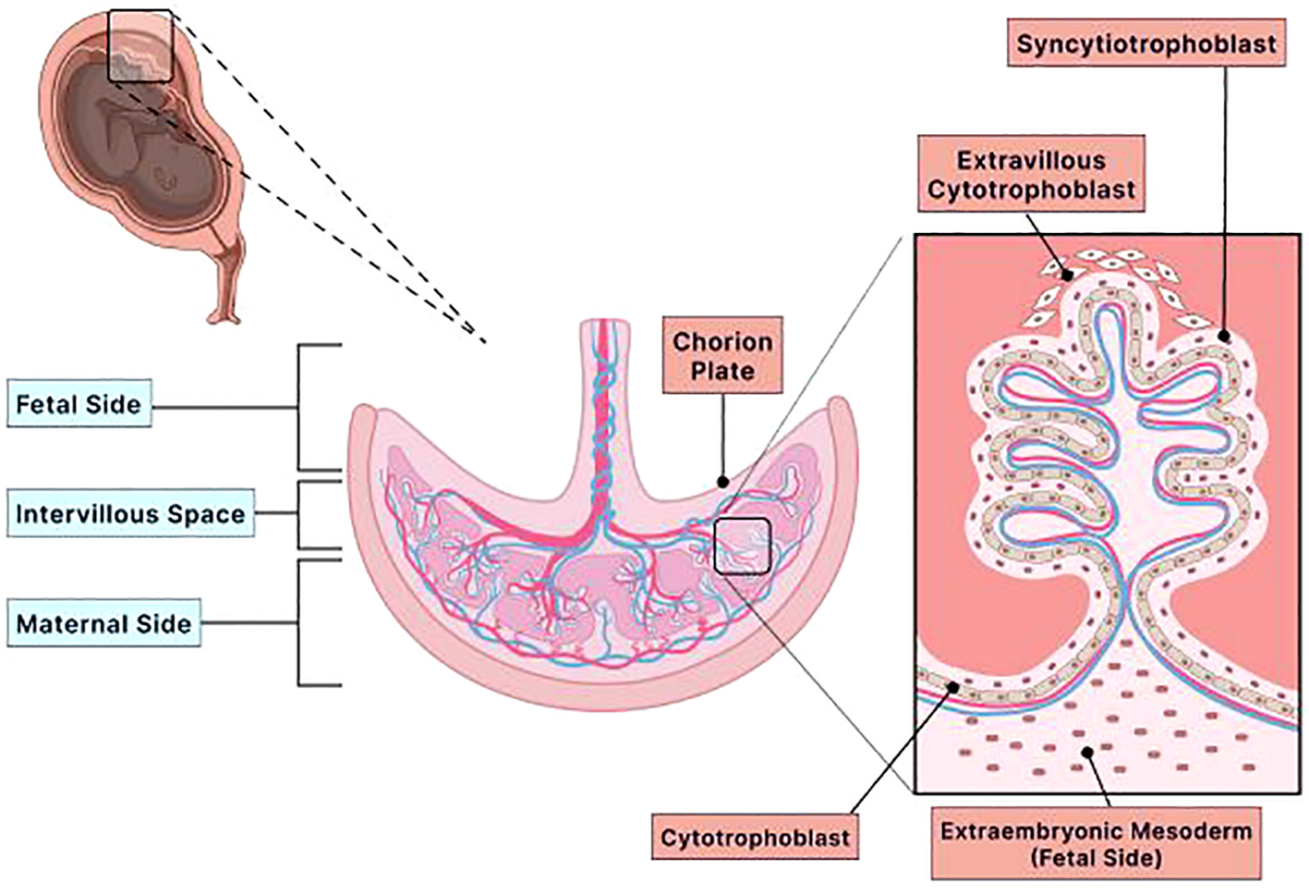
Structure of the placenta. The placenta is a multifaceted organ with many biological functions that are endocrinologic and immunologic. This temporary organ provides nutrients and oxygen, removes harmful waste products and carbon dioxide, and envelops the fetus in a protective immunologic membrane throughout gestation. Furthermore, the placenta has two distinct sides, fetal and maternal, and several layers and sublayers. The chorion layer is an immensely vascular membrane layer that surrounds the fetus. The chorion has two sublayers: the extraembryonic mesoderm and the trophoblast (cytotrophoblast and syncytiotrophoblast). Both layers are located on the fetal side of the placenta. The extraembryonic mesoderm is a tissue that contributes to the epithelium of the yolk sac, amnion, and chorionic villi (chorion plate). The trophoblasts are specialized cells crucial for embryo implantation because they interact with the maternal uterine environment to limit fetal immune rejection and ensure an adequate blood supply. The two types of trophoblast cells are cytotrophoblast and syncytiotrophoblast. Both cells cover the outer surface of the chorionic villus. The cytotrophoblast is the innermost lining of the chorionic villus, while the syncytiotrophoblast is the outermost lining of the chorionic villus that is bathed in maternal blood. Because the syncytiotrophoblast is bathed in maternal blood, it has the vital role of transporting nutrients to the fetus and removing waste. Therefore, these cells must have ample blood supply and blood flow and undergo apoptosis regularly to maintain adequate blood circulation. The cytotrophoblast cells are progenitor stem cells of the syncytiotrophoblast. These cells differentiate into the syncytiotrophoblast cells. Therefore, the cytotrophoblast has the specialized role of ensuring that the placenta has ample blood supply with nutrients and oxygen. Adequate blood supply and blood flow by the cytotrophoblast and syncytiotrophoblast are essential in maintaining the integrity of the placenta. If the integrity of the placenta becomes compromised, then adverse pregnancy complications, such as pre-eclampsia, preterm birth, and intrauterine growth restriction, will occur. Created with Biorender.com.

The placenta comprises two distinct sides, the maternal and the fetal sides (**Figure 1**). The chorion is a highly vascular outer embryonic membrane layer surrounding the fetus (14, 22). The chorion has two sublayers: the trophoblast (cytotrophoblast and syncytiotrophoblast) and the extraembryonic mesoderm (**Figure 1**) (15, 20). The trophoblast layer is positioned on the fetal side of the placenta. Trophoblast cells are essential for implantation because they interact with the maternal uterine endometrium, which promotes syncytiotrophoblast and cytotrophoblast development (20). Trophoblast differentiation and implantation ensure adequate blood supply and limit fetal immune rejection (16, 20). Cytotrophoblast cells are progenitor stem cells of the syncytiotrophoblast that can differentiate into syncytiotrophoblast cells as finger-like projections and ensure that the fetus receives an adequate blood supply with nutrients and oxygen (15, 20, 23). Syncytiotrophoblast cells transport nutrients to the fetus and remove waste products (16). This requires the syncytiotrophoblast to have ample blood supply and blood flow. In addition, these cells must undergo apoptosis continuously throughout gestation for the appropriate channeling of blood (24, 25). Because the syncytiotrophoblast cells have this specialized function and undergo apoptosis, these cells must be regenerated regularly to ensure adequate and continuous blood transport between the maternal and fetal circulations (26). A defect in trophoblast differentiation may compromise the integrity of the placenta, resulting in pregnancy complications, such as preterm birth, preeclampsia, and IUGR (27).

On the maternal side, the primary tissue that comprises this side of the placenta is the decidua, which originates from the endometrium (14). The decidua develops after the blastocyst attaches to the uterine wall, which involves tissue remodeling that supports both residential and immune cells (28). It includes terminally differentiated endometrial stromal cells, maternal blood, and maternal vascular cells (14, 16, 28). There are three distinct sections of decidua, which are named relative to the embryo: the decidua capsularis that covers the implanted embryo, the decidua basalis which is the region between the embryo and the myometrium, and the decidua parietalis, which lines the fetal membrane and the remaining endometrium of the placenta (14, 28, 29). Anchoring villi hold the chorion and the decidua together by securing the decidua basalis to the cytotrophoblast layer (14, 30). Like the trophoblast, the decidua promotes immune tolerance of the semi-allogenic (half of genes from the mother and half of genes from the father) fetus by limiting recognition from maternal immune cells. The decidua also provides nutritional support before placenta formation (28). Defects in the development of the decidua or decidualization may result in implantation failure, pregnancy loss, or pregnancy complications later in gestation (28).

### 2.3 Immune tolerance

The human placenta is complex and unique, allowing for an intimate contact between maternal and fetal cells throughout gestation (31). The developing fetus has both maternal and paternal antigens, to which the mother’s immune system recognizes the paternal antigens as foreign and leads to the activation of the maternal immune system (31). Therefore, a highly regulated immune system is needed between mother and child to create a beneficial immunological environment that protects the growing fetus from maternal–fetal tolerance, inflammation, and invading pathogens, such as viral infections (32, 33). The placenta is composed of various immune cells, such as natural killer T (NKT) cells, decidual natural killer (dNK) cells, T cells, dendritic cells, B cells, and macrophages (Hofbauer cells and decidual) (31–34). An immense network of cellular connections is formed in the placenta via the interaction of these immune cells, trophoblast cells, and decidual stromal cells to form the immune system in the placenta. An imbalance in this network allows pathogens to infect the placenta and cross the placental barrier to infect the fetus, along with pregnancy complications, such as preterm birth, preeclampsia, spontaneous abortion, and IUGR (33, 35).

## 3 Oxidative stress and ROS in the placenta

Oxygen is an essential element needed to sustain life. However, the presence of excessive oxygen or limited oxygen can lead to fatal toxicity of cells (36). The formation of ROS occurs as a natural byproduct of cellular oxidative metabolism resulting from the reduction of molecular oxygen generated by the mitochondria during oxidative phosphorylation (37). During mitochondrial oxidative phosphorylation, electrons transfer across respiratory chain enzymes and leak molecular oxygen (38). These electrons can leak prematurely and react with oxygen, producing ROS (38). ROS holds an oxygen atom with an unpaired electron in its outer shell. ROS are essential in regulating cell differentiation, cell signaling, cell differentiation, and inflammation-related factor production (39). However, considerable amounts of ROS at the molecular level can cause cellular and tissue damage, damaging nucleic acids, proteins, organelles, and membranes and inducing cell death or apoptosis (40, 41). Molecules and enzymes that are beneficial in reducing ROS and reducing the effects of ROS include antioxidants. Antioxidants inhibit oxidation and prevent or delay cell damage (42). The generation of ROS and antioxidants should be balanced to achieve homeostasis at the cellular and molecular levels. An imbalance between the formation of ROS and antioxidants leads to oxidative stress (42). This imbalance should be avoided for cellular processes to remain regulated.

Initially, at 0 to 9 weeks of pregnancy, the placenta develops in a state of low oxygen with an ambient pO_2_ <20 mmHg due to the blockage of maternal blood flow to the placenta by endovascular plugs of extravillous trophoblast (36, 43, 44). Previous studies have confirmed that pregnancies that are less than 10 weeks of gestation had no blood flow into the intervillous space, with *in vivo* measurements that indicate pO_2_ <20 mmHg (36, 43, 45). Therefore, a premature increase of oxygen tension within the first 10 weeks of gestation can lead to an increased risk of pregnancy loss, primarily due to the detrimental effects of ROS (46, 47). At 10 to 12 weeks gestation, the endovascular trophoblast plugs are lost, which allows maternal blood to perfuse in the intervillous space, thus increasing oxygen tension (36). Throughout pregnancy, the placenta adapts to the changing oxygen levels to support normal placental function by increasing the antioxidant defense within cells (48).

The placenta should remain homeostatic and balanced across gestation with no evidence of oxidative stress (5, 49, 50). Because the syncytiotrophoblast forms by fusion and differentiation of the cytotrophoblast, it must continuously undergo apoptosis to maintain homeostasis (15). Studies have shown that placental oxidative stress is a potent inducer of increased syncytiotrophoblast apoptosis through the mitochondrial oxidative pathway (51, 52). Increased programmed cell death of the syncytiotrophoblast disrupts the layer, resulting in homeostatic imbalance, which causes placental-derived material to be released into the maternal circulation, including tumor necrosis factor (TNF-alpha) and syncytiotrophoblast microparticles (SBTM) (51). Placental trophoblast and endothelial cells form the barrier separating the maternal and fetal circulations (53). Oxidative stress in these cells can lead to the rupture of this barrier and reduce placenta oxygenation or the intermixing of the maternal–fetal circulations (16, 54). Therefore, low levels of ROS are necessary for syncytiotrophoblast formation. In contrast, an imbalance between the ratio of ROS and antioxidants adversely downregulates the syncytiotrophoblast due to increased apoptosis (4, 37, 52).

## 4 Melatonin in the placenta

### 4.1 Melatonin and the circadian rhythm in the placenta

Every living organism and nearly every organ has a circadian clock that governs the daily rhythmicity of several physiological and biological processes (55). Core clock genes control the circadian rhythm in the suprachiasmatic nucleus (SCN), the master central pacemaker in the hypothalamus (34, 56). The SCN regulates the photoperiodic programming of the daily circadian clock and coordinates the clock machinery in peripheral tissues (57, 58). The cellular clock oscillates through core clock genes depending on the time of day. Through a transcriptional/translational feedback loop, the heterodimer *BMAL1/CLOCK* activates the heterodimer *Per/Cry* expression that suppresses the transcription of *BMAL1* and *CLOCK* (59–61). Another short feedback loop involves the participation of *BMAL1/CLOCK* to activate the expression of *REV-ERBα* and *RORα* through binding to the nuclear orphan receptor (*ROR*) at the promoter site (34, 62).

The uterus and the placenta utilize circadian rhythm to carry out certain physiological functions, e.g., hormone release, parturition, and immune function, and entrain the fetus’ circadian rhythm (2, 63–66). Studies have shown that melatonin can synchronize the clock machinery in healthy and damaged cells to upregulate or downregulate specific clock genes to sustain optimal physiology in cells (34, 67, 68). Moreover, the placenta employs a circadian rhythm for the rhythmic release of melatonin. Melatonin is a ubiquitous molecule that conducts many functions. It is mainly known for its role as one of the circadian rhythm regulators, primarily synthesized in the pineal gland by pinealocytes under dark conditions (34, 69). During darkness, melatonin is rhythmically produced by the pineal gland—concentrations peak between the hours of 2 a.m. and 4 a.m (8).

**Figure 2:**
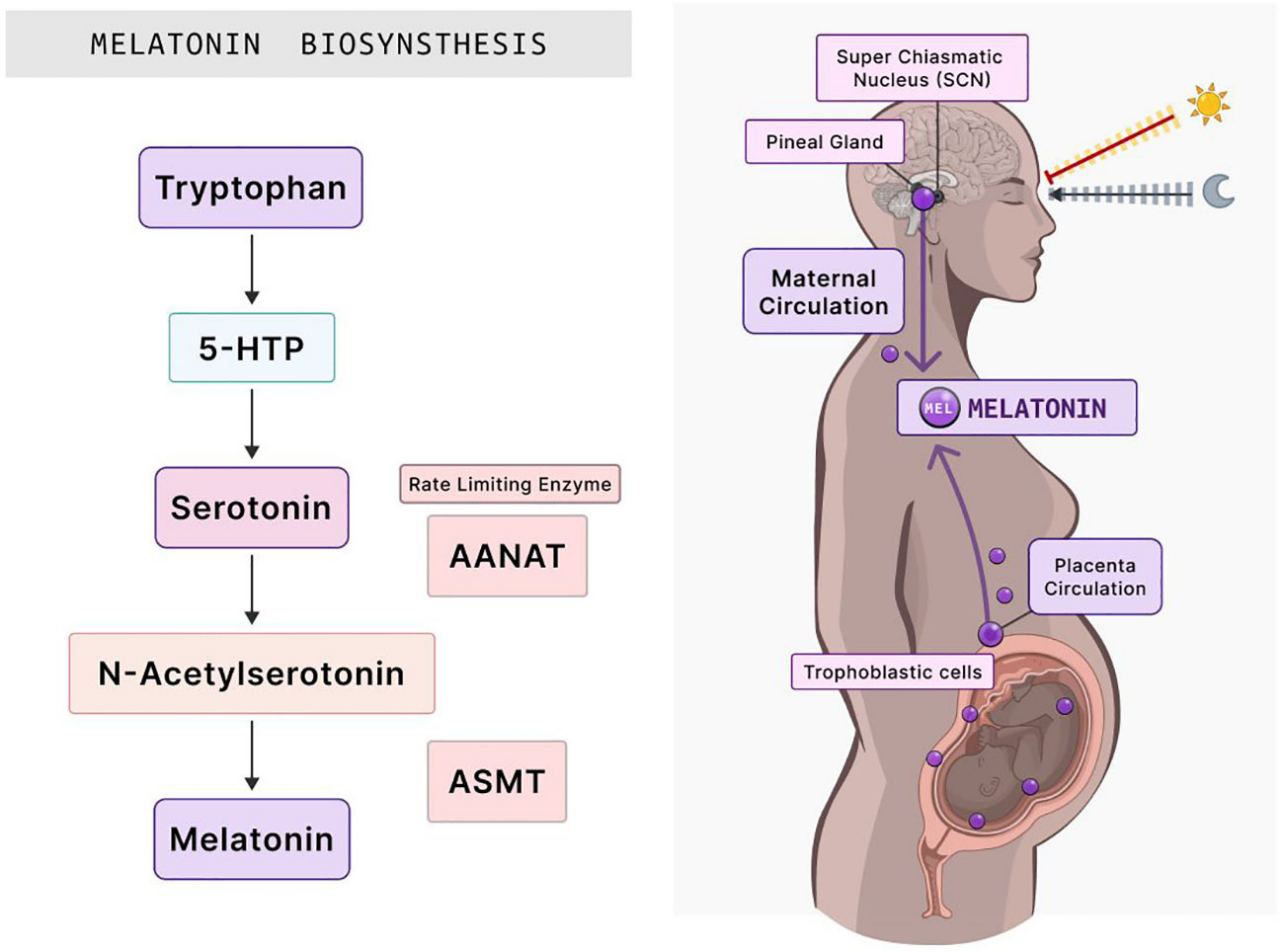
Melatonin biosynthesis and its distribution in the human body during pregnancy. The synthesis of melatonin begins with the amino acid tryptophan, which subsequently transforms into 5-HTP. This intermediate is then converted into serotonin. The rate-limiting enzyme, AANAT, acts on serotonin, culminating in N-acetylserotonin production. ASMT then catalyzes the final step, yielding melatonin. Once synthesized, the SCN in the brain, which regulates circadian rhythms, signals the pineal gland to release melatonin into the maternal circulation. Notably, melatonin is also synthesized within the placenta by trophoblastic cells. This melatonin subsequently influences various processes in placental function, essential for fetal growth and development. 5-HTP, 5-hydroxytryptophan; AANAT, arylalkylamine N-acetyltransferase; ASMT, acetylserotonin O-methyltransferase; SCN, suprachiasmatic nucleus. Created with Biorender.com.

Melatonin (N-acetyl-5-methoxytryptamine) is synthesized from serotonin (5-hydroxytryptamine) through a two-step reaction (**Figure 2**). In the first step, serotonin becomes acetylated by arylalkylamine N-acetyltransferase (AANAT) to become N-acetyl serotonin, the rate-limiting step. AANAT is the rate-limiting enzyme of melatonin because it controls the circadian rhythm of melatonin production via the pineal gland. The second step involves N-acetyl serotonin becoming methylated by acetyl serotonin o-methyltransferase (ASMT) to become melatonin (70–72). Given that serum melatonin concentrations are higher in pregnant than in non-pregnant women, studies have indicated that the primary source of this melatonin is the placenta (8–10). How the placenta produces melatonin without the need for the pineal gland or whether the circadian rhythm plays a role in this production has to be studied. The need for melatonin in the placenta is crucial for both the mother and the fetus because it depends on placental melatonin and maternal serum melatonin to provide photoperiodic information to control the internal rhythms of the fetus (34, 64). The placenta exposes maternal melatonin to the fetus at daily rhythmic intervals, with low concentrations during the day and high concentrations at night (8). Disrupting this rhythm during pregnancy may lead to detrimental outcomes for the mother and the growing fetus *in utero* and in adult life (8, 56, 61, 73). The development of the embryo, uterine implantation, placentation, and delivery may be regulated by the clock molecular machinery in the circadian rhythm (73). Circadian disruption or chronodisruption in pregnant women can lead to adverse outcomes in the offspring (61). Maternal chronodisruption is caused by mistimed eating, shift work, traveling across time zones, and immoderate artificial light exposure at nighttime (61). The impairment of the circadian rhythm during pregnancy compromises melatonin production, inhibiting the rhythm of melatonin release (74, 75). Chronodisruption may promote chronic illnesses in postnatal life, including diabetes, obesity, and cardiovascular disease (61).

### 4.2 Melatonin receptors in the placenta

**Figure 3:**
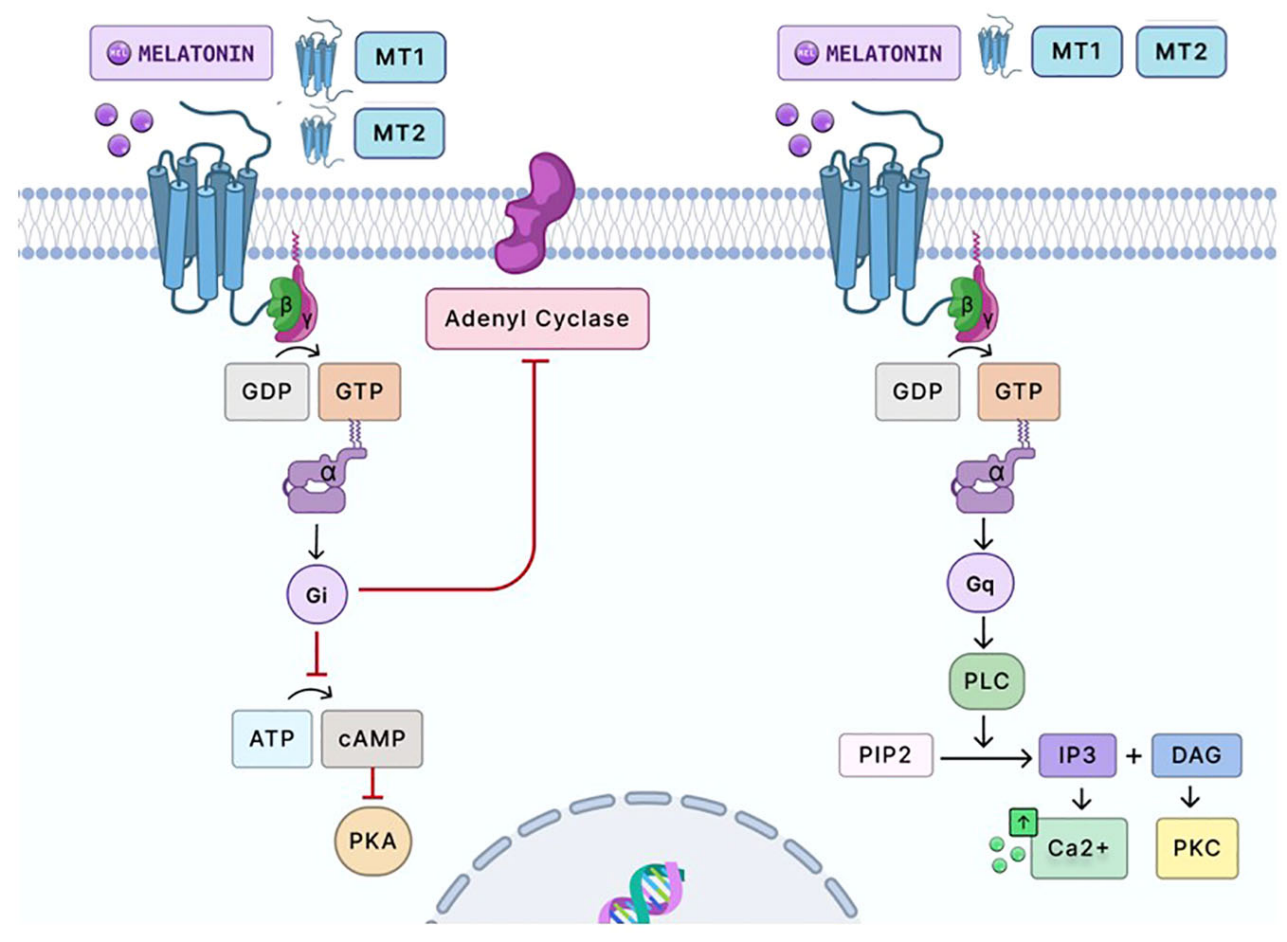
Signaling pathway of melatonin via G-protein-coupled receptors. Melatonin primarily signals through two G-protein-coupled receptors, MT1 and MT2. Upon melatonin binding, the MT1 homodimer receptor and MT2 homodimer receptor couples with Gi proteins, inhibiting adenylate cyclase. This results in decreased cAMP production and subsequent reduced activation of PKA. MT1 and MT2 heterodimer activation can also couple with Gq/11 proteins to stimulate PLC. This enzyme catalyzes the conversion of PIP2 into IP3 and DAG. IP3 subsequently promotes calcium release from intracellular stores, elevating intracellular calcium levels. Simultaneously, DAG and calcium can activate PKC, which influences a broad spectrum of cellular activities. PKC modulates numerous cellular processes by phosphorylating target proteins. The heterodimerization of MT1 and MT2 in the placenta has yet to be studied. However, the binding of melatonin on the MT1 homodimer and MT2 homodimer has been shown to elicit antioxidant and anti-inflammatory responses in placental cells by reducing oxidative stress and inflammation. Moreover, activating these receptors in these cells indicates autocrine and paracrine activity, imperative to placental homeostasis and fetal development. Gi and Gq, G-proteins; GDP and GTP, guanosine diphosphate and guanosine triphosphate, respectively; ATP, adenosine triphosphate; cAMP, cyclic adenosine monophosphate; PKA, protein kinase A; PLC, phospholipase C; PIP2, phosphatidylinositol 4,5-bisphosphate; IP3, inositol 1,4,5-trisphosphate; DAG, diacylglycerol; Ca^2+^, calcium ions; PKC, protein kinase C. Created with Biorender.com.

Melatonin’s effects are mediated by two receptors, MT1 (Mel1a) and MT2 (Mel1b) (**Figure 3**) (76, 77). The MT1 receptor is approximately 350 amino acids long, while the MT2 receptor is 362 amino acids long (78). The molecular weight of both receptors is about 39–40 kDa (78). MT1 and MT2 are G-protein coupled receptors. G-protein coupled receptors are essential membrane proteins that form the fourth-largest superfamily in the human genome (79). The G-protein receptor has three subunits: α, β, and γ. When a ligand binds to the extracellular receptor, this initiates a signal transduction cascade that induces a conformational change, promoting the activation of the heterotrimeric GTP-binding protein (G-protein) (79). As the G-protein coupled receptor becomes activated, the α subunit disassociates from the β and γ subunits and releases guanine diphosphate (GDP). It binds to guanine triphosphate (GTP), which results in the conformational changes that later trigger the signal transduction of different G-proteins: inhibitory G-protein (G_i_), stimulatory G-protein (G_s_), and guanine-nucleotide binding protein (G_q_) (79). Each of these proteins has different functions that activate distinct signaling pathways. The G_s_ protein stimulates the increase of cyclic AMP (cAMP) levels by activating adenylyl cyclase, while G_i_ inhibits cAMP levels (79). Moreover, G_q_ induces the expression of phospholipase C, which stimulates protein kinase C (PKC) and calcium (79). Consequently, the MT1 receptor homodimer and MT2 homodimer bind and activate the Gi–G protein and inhibits adenyl cyclase pathway signaling, decreasing forskolin-stimulated cAMP and protein kinase A signaling (60, 77, 80, 81). The MT1 and MT2 receptor heterodimer is associated with G_q_, which enables the production of protein kinase C (PKC) and the increase of calcium associated with IP_3_ (60, 77, 80, 81). MT1 and MT2 are widely distributed throughout the body and are present in almost all human cells (72, 82, 83). The primary responsibility of the MT1 receptor involves regulating the circadian cycle, while MT2 receptors control the body temperature through the peripheral tissue. In both the cytotrophoblast and the syncytiotrophoblast, studies have detected the presence of melatonin receptors in both primary isolated cells of the placenta and placental trophoblast cell lines (JEG-3 and BeWo) (34, 72, 82). The placenta constantly synthesizes melatonin receptors to promote the survival of the cytotrophoblast by decreasing free radicals and angiogenesis (34). Moreover, when disease (preeclampsia and preterm birth) is present in the placenta, melatonin receptor activity and melatonin levels are both decreased (34, 84). The binding of melatonin on MT1 homodimers and MT2 homodimers and the activation of the receptors indicate autocrine and paracrine activity needed for placental homeostasis and fetal development (34).

### 4.3 Melatonin in the immune microenvironment of the placenta

Pregnancy is associated with immunological changes that result in the fetus avoiding immune rejection by the mother. Increasing evidence associates immunological changes in pregnancy with melatonin and the circadian clock and synchronous programming of the immune system (34, 85). Melatonin possesses immunomodulatory properties (85) that use circadian regulation to stimulate cytokine production and lymphocyte proliferation and enhance phagocytosis (85–90). In both the luteal phase and early gestation, uterine NK cells represent most of the lymphocyte population. The decidual NK cells CD56^dim^ and CD56^bright^ comprise most of the leukocyte population in the decidua (91). Decidual cells activate thymocytes, macrophages, neutrophils, and NK cells (34, 92). At the same time, prostaglandins aid in uterine contractions and amplify TNF-*α* and IL-6 release (93). Alterations in NK cells, T regulatory cells (Tregs), Th17, and Th1/Th2 ratio may be associated with pregnancy-related complications, pregnancy loss, implantation failure, and preeclampsia (92). Melatonin affects the T cell subpopulation in gestation by increasing the differentiation of Th17 and Tregs (94, 95). Melatonin rhythm may be synchronized with Th1/Th2 cell populations in protecting and maintaining fetal survival (96).

Melatonin levels have a significant role in immunity during gestation by enhancing innate and cellular immunity. In the first trimester of pregnancy, macrophages are the second most abundant leukocyte population in the decidual layer of the placenta (33). They have the prominent role of sensing pathogens and immune effector cells, which suggests a central role in the inflammatory response of both placental and decidual infections (97). Melatonin activates the progenitor cell production of macrophages, NK cells, and granulocytes (98). Furthermore, melatonin promotes immune homeostasis during late and early pregnancy (34, 97). It can modify many transcription factors in macrophages, including hypoxia-inducible factor (HIF-1 *α*), nuclear factor-k-B binding (NF-kB), and interferon regulatory factors (IRFs) (99). In addition, melatonin inhibits cytokine expression in cells treated with lipopolysaccharide (LPS) (100).

### 4.4 Role of melatonin as an antioxidant and anti-inflammatory in the placenta during oxidative stress

Melatonin has a wide array of functions due to its ubiquitous nature. In reproductive physiology, melatonin exerts endocrine, autocrine, intracrine, and paracrine effects in the placenta (1, 8, 101). Melatonin can pass through the placental barrier and enter the fetal circulation to promote fetal growth and entrain the circadian rhythm of the fetus (34, 69). During pregnancy, melatonin levels increase throughout gestation and peak at term, promoting placental trophoblast cell survival and homeostasis and regulating hormonal production (102). Besides being a circadian-regulating hormone, melatonin is a powerful antioxidant and anti-inflammatory that can offset the effects of free radicals and scavenge ROS (103, 104). Melatonin can act directly on the free radicals or indirectly by stimulating antioxidant enzymes through its receptors (103, 105). Melatonin is both hydrophilic and lipophilic, which means that it can maneuver between cellular compartments easily and has access to ROS produced by the mitochondria (106, 107). In the presence of oxidative stress, melatonin can protect placental trophoblast cells against damage (71, 108). Melatonin decreases ROS levels by neutralizing and increasing the antioxidant enzymes’ (superoxide dismutase, glutathione peroxidase, glutathione reductase, and catalase) expression and activity (104, 109, 110). Moreover, melatonin can protect trophoblasts from apoptosis when oxidative stress occurs (108, 111), preventing pregnancy complications. In immunocompromised pregnancies that have rendered pregnancy complications, a supraphysiological dosage of melatonin has been shown to reverse the adverse effects of these complications for both the mother and the fetus (2, 34, 86).

Dysfunction of the placenta and oxidative stress contribute to unfavorable conditions in pregnancy, such as pre-eclampsia and preterm birth (2, 4, 24, 50, 112). Preterm birth is characterized as parturition that occurs before 37 weeks of gestation and is commonly associated with intrauterine infection and inflammation (2, 3, 113). A recent study found that melatonin treatment offsets placental inflammation in offspring exposed to LPS-induced maternal inflammation (84). Preeclampsia occurs after 20 weeks of pregnancy and is associated with hypertension and proteinuria, which induces oxidative stress in placental cells (3, 5, 114, 115). A study noted reduced serum melatonin levels and MT1 receptors in preeclamptic women (116). This reduction may have been due to the decreased placental secretion of melatonin induced by preeclampsia (116). Moreover, another study by Sagrillo-Fagundes and his group found that melatonin significantly regulated autophagy and inflammation in primary trophoblast cells that were exposed to a hypoxic/reoxygenation environment, mimicking preeclamptic pregnancy, thus reducing apoptosis in these cells. This study suggests that the placenta’s endogenous melatonin production is inhibited during preeclampsia, thus limiting melatonin in maternal blood and the placenta (111). Additionally, melatonin has been shown to reverse other complications during pregnancy and maintain a healthy pregnancy. In a study in mice, melatonin improved the efficiency of the placenta by increasing the expression of antioxidant enzymes in undernourished pregnancies (117). In a similar study, gestating sows were supplemented with melatonin, which indicated that melatonin could improve the redox status of the mother, fetus, and placenta and amplify the placental growth and function. This may be primarily due to melatonin’s ability to enhance the placenta’s antioxidant status and inflammatory response and activation (118). In all of these studies, melatonin had the potential benefit of acting as a preventative therapeutic agent for preterm birth, preeclampsia, and other pregnancy complications (84, 101, 116). In future studies, it will be essential to understand how melatonin uses its anti-inflammatory and antioxidant properties to target inflammation and oxidative stress in the placenta.

The induction of oxidative stress and inflammation at the maternal–fetal interface leads to activation of the NLRP3 inflammasome (119–121). The NLRP3 inflammasome complex is an intermediary in innate immune signaling that contributes to the pathogenesis of several diseases (122). Therefore, inhibition of the NLRP3 inflammasome pathway is a potential therapeutic target for inflammatory-related disorders (120, 122). The formation of the NLRP3 inflammasome is triggered by both internal and external factors, such as pathogen-associated molecular patterns (PAMPs), danger/damage-associated molecular patterns (DAMPs), and ROS (121, 123). An inducer of NLRP3 activation is the disassociation of thioredoxin-interacting protein (TXNIP), an intracellular redox regulator that inhibits antioxidants, from TRX (thioredoxin), a redox protein, via ROS or oxidative stress (122, 124). TXNIP and TRX form a complex with a redox relationship, which neutralizes the inhibitory properties of TXNIP, such as over-accumulation of ROS. After TXNIP disassociates and binds to NLRP3, it activates inflammasome (122, 124). The activation of the inflammasome complex entails two consecutive signals, namely (1): the priming signal is established by the activation of TLR receptors (PAMPs) through (for example) LPS, which leads to the secretion of inflammatory cytokines by the nuclear translocation of NF-kb (122, 123, 125, 126) and (2) the activation signal occurs due to the stimulation and binding of several factors that the complex recognizes, such as ROS and TXNIP/TRX disassociation, leading to the generation of caspase-1 (122, 123, 125, 126). Melatonin induces the inhibitory function of the NLRP3 inflammasome by either inhibiting or activating several different proteins and pathways (122). One of the several proteins and pathways melatonin uses to inhibit NLRP3 activation is NF-kb signaling, a master regulator of the priming phase of NLRP3 inflammasome activation (34). Melatonin also reduces TXNIP, which leads to the suppression of ROS and NLRP3 (122). Lastly, melatonin upregulates s Nrf2, an antioxidant protein that promotes ROS scavenging and elimination to facilitate protection against NLRP3 inflammasome activity through ROS scavenging and elimination mediated by Nrf2 (102).

## 5 Viral infections during pregnancy

**Figure 4:**
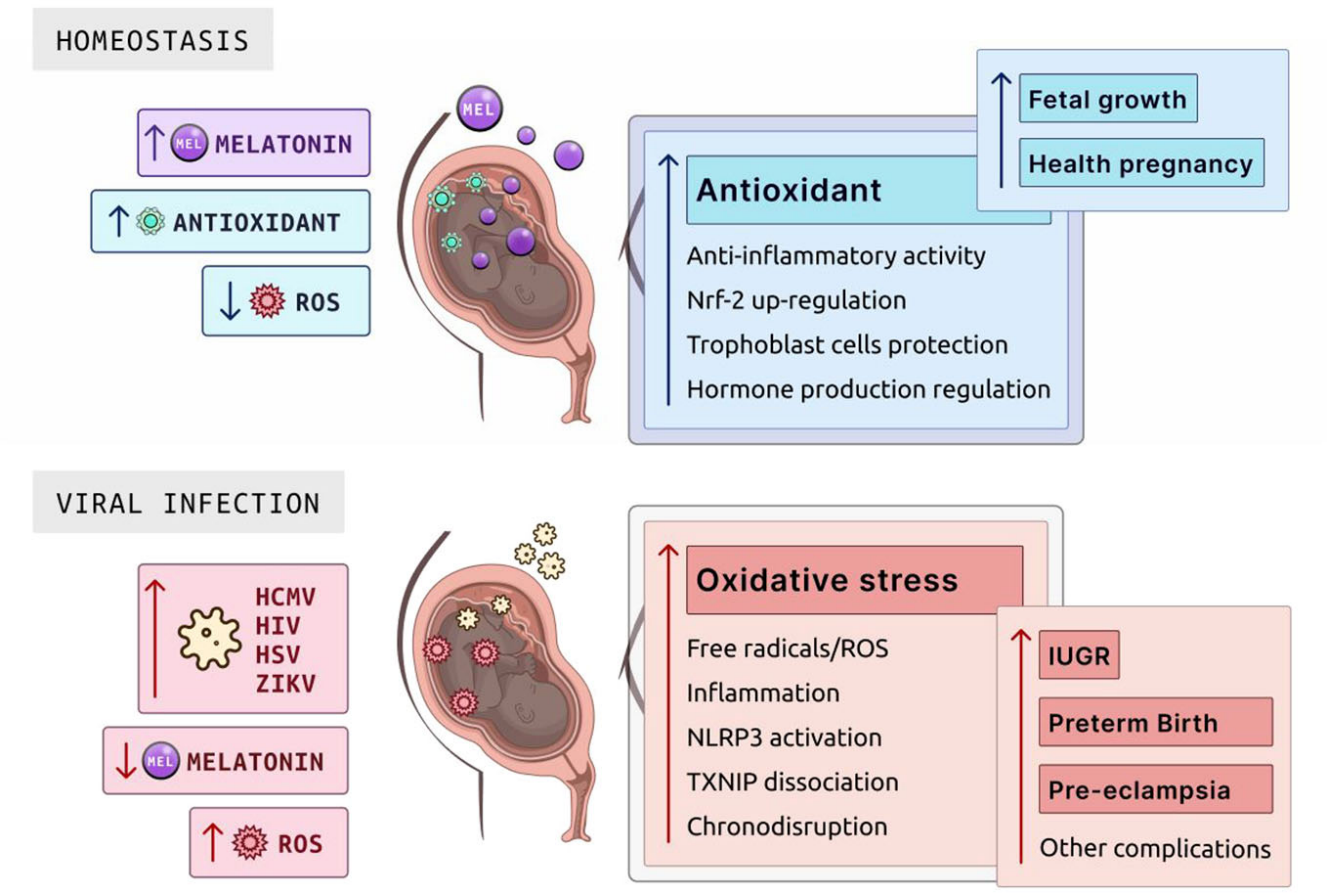
Role of melatonin in homeostasis and viral infection during pregnancy. Melatonin is pivotal in maintaining cellular homeostasis due to its antioxidant properties, which neutralize ROS. In the placenta, melatonin supports a healthy pregnancy through its antioxidant activity, anti-inflammatory actions, Nrf-2 upregulation to enhance cellular defense mechanisms, trophoblast cell protection essential for placental and fetal development, and hormone production regulation to balance the hormonal environment for fetal growth. In the context of viral infections such as HCMV, HIV, HSV, and ZIKV, melatonin levels drop significantly, leading to an increase in ROS. This surge in oxidative stress can cause complications, including free radicals/ROS production, NLRP3 activation, inflammation, TXNIP dissociation affecting cellular stress responses, and chronodisruption, which together elevate the risks of IUGR, preterm birth, pre-eclampsia, and other potential complications. ROS, reactive oxygen species; HCMV, human cytomegalovirus; HIV, human immunodeficiency virus; HSV, herpes simplex virus; ZIKV, Zika virus; IUGR, intrauterine growth restriction; NLRP3, NOD-, LRR-, and pyrin domain-containing protein 3; TXNIP, thioredoxin-interacting protein. Created with Biorender.com.

Inflammation at the maternal–fetal interface puts the developing fetus at risk for IUGR (127). Placental inflammation can induce oxidative stress, resulting in an overproduction of free radicals/ROS and reduced antioxidant ability (**Figure 4**) (113). Furthermore, when a mother either contracts a viral infection during pregnancy or acquires a viral infection before pregnancy, this can cause an inflammatory cascade at the maternal–fetal interface (115, 127–129). Some viral infections can cross the placental barrier and influence viral-mediated inflammatory oxidative stress on placental trophoblast cells (127, 130). These viruses include human cytomegalovirus, HIV, herpes simplex virus, and Zika virus (115, 127, 130). These viruses can transmit vertically to the fetus (131, 132). Viral activation in pregnancy is associated with releasing inflammatory chemokines and cytokines in the placenta, enhancing ROS generation in immune and trophoblast cells and resulting in oxidative stress (**Figure 4**). Viral activation can also cause the syncytiotrophoblast cells to be more susceptible to apoptosis at a much faster rate (26).

The circadian rhythm and CLOCK genes are essential in regulating the reproductive system (73, 133) and parturition (56). Misalignment of this rhythm (chronodisruption) can adversely affect reproductive function and birthing outcomes (73). A potential link between disruption of the circadian rhythm and viral infections suppressing the immune system has been suggested by several investigators (113, 115, 134, 135). Viruses can rework the biological processes of the infected host to accelerate replication in the body. An interchange between host immunity, virus, and biological clock may influence disease outcomes (134)—the mechanism of this in the placenta has yet to be widely studied. However, evidence demonstrates how viral-induced inflammation can incite chronodisruption to inhibit melatonin and induce oxidative stress and inflammation in the placenta (2, 51, 71, 113, 115, 128, 136–138). A therapeutic strategy with melatonin during pregnancy may be plausible to increase physiological melatonin depletion due to chronodisruption and viral infection.

Melatonin’s pro-apoptotic, antioxidant, and anti-inflammatory properties can alleviate inflammatory-mediated oxidative stress by neutralizing and scavenging ROS. However, the clinical capabilities of melatonin to relieve inflammatory oxidative stress caused by a viral infection in the placenta have yet to be elucidated as a therapeutic. In a healthy pregnancy, there is a high concentration of serum melatonin in maternal blood and the placenta (9). Melatonin can alleviate oxidative stress by keeping a homeostatic balance of ROS and antioxidants at the maternal–fetal dyad (**Figure 4**) (9, 51, 138). The placenta is a crucial source of oxidative stress, and this homeostatic imbalance can lead to inflammatory disorders like preterm birth and preeclampsia (3, 49–51). Furthermore, melatonin can use its anti-inflammatory properties to directly target inflammatory pathways within the innate immune system to reduce inflammation and oxidative stress. Examples of inflammatory pathways include the TLR/NLRP3 pathway, where melatonin upregulates Nrf2 to inhibit NF-kb signaling, inhibiting NLRP3 and cytokine release (139, 140).

## 6 Conclusion

This article has reviewed melatonin’s beneficial properties as an antioxidant and anti-inflammatory in oxidative stress and inflammation in the placenta. Melatonin can be a potential novel therapeutic for oxidative stress-induced pregnancy complications like preeclampsia and preterm birth. More clinical studies are needed to analyze and observe melatonin in pregnant women and characterize its impact on placental cells in potentially reducing inflammatory-mediated oxidative stress.

### 6.1 Future directions

Although there is no strong evidence currently to corroborate melatonin as a powerful antioxidant and anti-inflammatory in the placenta during oxidative stress, inflammation, and viral infection, future work should address whether melatonin’s antioxidant properties in the placenta are due to direct scavenging of ROS (supraphysiological) or receptor-mediated (physiological). Moreover, future work should address whether melatonin increases Nrf2 to deactivate the NLRP3 inflammasome during a viral infection at the maternal–fetal interface. Lastly, potential studies should determine safe and effective dosages that can be given to pregnant women during adverse pregnancy events like preeclampsia.

## Author contributions

TJ: Conceptualization, Investigation, Writing – original draft, Writing – review & editing, Visualization. VS: Visualization, Writing – review & editing. DH: Writing – review & editing. RC: Writing – review & editing. EJ: Conceptualization, Funding acquisition, Supervision, Writing – review & editing.
